# Austin District Medical Society

**Published:** 1890-09

**Authors:** F. R. Martin, T. J. Bennett

**Affiliations:** President; Secretary


### Society Notes.

### AUSTIN DISTRICT MEDICAL ASSOCIATION.

This Society will meet in Medical Hall, Austin, September 25, 1890. The following papers will be read:

"A Case of Traumatic Tetanus," by S. G. Harper, M. D.; discussion J. O. Lewright, M. D. and W. A. Ellison, M. D.

"Some of the Delusions of the Insane," by John Preston, M. D.; discussion opened by A. N. Denton, M. D., and F. A. Maxwell, M. D.

"The Treatment of Epithelioma of Face by Caustics," by A. C. Conner, M. D.; discussion opened by W. M. Cunningham, M. D., and T. D. Wooten, M. D.

"Antipyretics," by R. S. Gregg, M. D.; discussion opened by J. W. McLaughlin, M. D., and C. O. Weller, M. D.

"Eclampsia," by R. Atkinson, M. D.; discussion by R. M Swearingen, M. D., and Ralph Steiner, M. D.

Election of officers will be held.

Every physician accessible to Austin and eligible to membership in the State Association, is eligible to membership in the Austin District Medical Society, and should avail himself of the privileges of this flourishing order without delay. The time has come in Texas when every regular physician must belong to a good working society, and must work, or he will be classed where he natually belongs, among the non-progressive, unreliable irregulars.

You will observe we have a good programme, and will have an interesting session. You are cordially invited to attend and take part in the proceedings.

F. R. MARTIN, President.

T. J. BENNETT, Secretary.

